# Net, excess and absolute adsorption in mixed gas adsorption

**DOI:** 10.1007/s10450-017-9875-4

**Published:** 2017-02-24

**Authors:** Stefano Brandani, Enzo Mangano, Mauro Luberti

**Affiliations:** Scottish Carbon Capture and Storage, School of Engineering, The University Edinburgh, The King’s Buildings, Mayfield Road, Edinburgh, EH9 3FB UK

**Keywords:** Adsorption equilibria, Net adsorption, Absolute adsorption, Ideal adsorbed solution theory

## Abstract

The formulation of a thermodynamic framework for mixtures based on absolute, excess or net adsorption is discussed and the qualitative dependence with pressure and fugacity is used to highlight a practical issue that arises when extending the formulations to mixtures and to the Ideal Adsorbed Solution Theory (IAST). Two important conclusions are derived: the correct fundamental thermodynamic variable is the absolute adsorbed amount; there is only one possible definition of the ideal adsorbed solution and whichever starting point is used the same final IAST equations are obtained, contrary to what has been reported in the literature.

**Electronic supplementary material:**

The online version of this article (doi:10.1007/s10450-017-9875-4) contains supplementary material, which is available to authorized users.

## Introduction

In general it is not possible to measure gas adsorption directly and this has led to the use of excess (see for example Sircar 1999) and net adsorption (Gumma and Talu 2010), which are valid options when reporting experimental data but not to develop a thermodynamic framework (Myers and Monson 2014) which has to be based on absolute adsorption. Talu (2013) has recently developed the use of net adsorption within a thermodynamic framework and has derived a version of the Ideal Adsorbed Solution Theory (IAST) without the need to convert this to absolute adsorption.

We have recently discussed the definitions of adsorption for pure gases (Brandani et al. 2016) in microporous solids and in this contribution we extend this to the case of mixed gases. This further analysis highlights some important qualitative characteristics which can be used to show that the IAST formulation of Talu (2013) is inconsistent. The second part of this contribution will therefore try to resolve this inconsistency and show that there is only one possible definition of an ideal adsorbed phase.

## Definitions of net, excess and absolute adsorption for mixed gases

We consider here the system to be that of a rigid microporous crystal as assumed by Myers and Monson (2014) as adopted by Brandani et al. (2016). We are interested in adsorbents for separation processes, where the micropore volume is well defined and the adsorption on the external surface is negligible by comparison.

We are defining a fixed volume, *V*_*S*_, which comprises the porous solid and the micropore volume. We can define the total number of moles in the system as

1 $$n_{{Tot}} = \mathop \sum \limits_{i} n_{i}^{A} + n^{S}$$

where the suffix A indicates an adsorbate and S is the solid.

In absolute adsorption we simply remove the solid and define

2 $$n_{Tot}^{abs}=n_{Tot}^{{}}-{{n}^{S}}=\underset{i}{\mathop \sum }\,n_{i}^{A}~$$

In net adsorption we subtract from this the moles that would be in a fluid at the same pressure and temperature of the system with a concentration at equilibrium with the adsorbed phase that would occupy the volume of the system.

3 $$n_{Tot}^{net}=n_{Tot}^{abs}-{{V}_{S}}\underset{i}{\mathop \sum }\,{{c}_{i}}=\underset{i}{\mathop \sum }\,\left( n_{i}^{A}-{{V}_{S}}{{c}_{i}} \right)=\underset{i}{\mathop \sum }\,n_{i}^{net}~~$$

In the case of a mixture it is a bit more complicated to define the excess amount adsorbed, since different molecules may access different portions of the micropore volume. Nevertheless, if one can define a reference non accessible volume for the excess adsorbed amount, then excess adsorption can be defined. This is obtained by subtracting the moles that would be in a fluid at the same pressure and temperature of the system with a concentration at equilibrium with the adsorbed phase that would occupy the accessible volume of the system.

4 $$n_{{Tot}}^{{ex}} = n_{{Tot}}^{{abs}} - \left( {V_{S} - V_{{NA}} } \right)\mathop \sum \limits_{i} c_{i} = \mathop \sum \limits_{i} \left[ {n_{i}^{A} - \left( {V_{S} - V_{{NA}} } \right)c_{i} } \right] = \mathop \sum \limits_{i} n_{i}^{{ex}}$$

In Eqs. 3 and 4 the total concentration can be written in terms of the compressibility factor, *z*, which is equal to one for an ideal gas.

5 $$\underset{i}{\mathop \sum }\,{{c}_{i}}=\frac{P}{zRT}$$

We can now define the adsorbed phase concentrations by dividing the number of moles by the volume

6 $$q_{{Tot}}^{{abs}} = \frac{{n_{{Tot}}^{{abs}} }}{{V_{S} }} = \mathop \sum \limits_{i} \frac{{n_{i}^{A} }}{{V_{S} }} = \mathop \sum \limits_{i} q_{i}^{A}$$

and the equivalent net and excess concentrations

7 $$q_{Tot}^{net}=\frac{n_{Tot}^{abs}}{{{V}_{S}}}-\underset{i}{\mathop \sum }\,{{c}_{i}}=\underset{i}{\mathop \sum }\,q_{i}^{A}-\underset{i}{\mathop \sum }\,{{c}_{i}}~~~$$

8 $$q_{Tot}^{ex}=\frac{n_{Tot}^{abs}}{{{V}_{S}}}-{{\varepsilon }_{m}}\underset{i}{\mathop \sum }\,{{c}_{i}}=\underset{i}{\mathop \sum }\,q_{i}^{A}-{{\varepsilon }_{m}}\underset{i}{\mathop \sum }\,{{c}_{i}}~~~$$

As discussed by Brandani et al. (2016) the correct limit at low pressure is given by Henry’s law and for mixed gases

9 $$q_{Tot}^{abs}=\underset{i}{\mathop \sum }\,{{K}_{i}}{{c}_{i}}~~~~~$$

10 $$q_{Tot}^{net}=\underset{i}{\mathop \sum }\,\left( {{K}_{i}}-1 \right){{c}_{i}}~~~~~$$

11 $$q_{Tot}^{ex}=\underset{i}{\mathop \sum }\,\left( {{K}_{i}}-{{\varepsilon }_{m}} \right){{c}_{i}}~~~~~$$

while at infinite pressure

12 $$q_{\infty }^{abs}=\frac{\eta _{CP}^{A}}{{{\eta }_{CP}}}\frac{{{V}_{S}}-{{V}_{NA}}}{{{V}_{S}}}\underset{i}{\mathop \sum }\,c_{i}^{\infty }=~q_{Sat}^{abs}~$$

ie the saturation capacity of the micropores and

13 $$q_{\infty }^{net}=\left( \frac{\eta _{CP}^{A}}{{{\eta }_{CP}}}\frac{{{V}_{S}}-{{V}_{NA}}}{{{V}_{S}}}-1 \right)\underset{i}{\mathop \sum }\,c_{i}^{\infty }<0~$$

14 $$q_{\infty }^{ex}=\left( \frac{\eta _{CP}^{A}}{{{\eta }_{CP}}}-1 \right){{\varepsilon }_{m}}\underset{i}{\mathop \sum }\,c_{i}^{\infty }<0~$$

Therefore qualitatively the absolute adsorbed amount will increase monotonically to the saturation capacity. The net and excess adsorbed amounts will initially increase and then go through a maximum and the correct limit at infinite pressure will be negative and finite if an equation of state is used which has the correct limit of infinity for the compressibility factor (Brandani and Brandani 2007) and hence a finite density.

For the excess adsorbed amount, one alternative approach to defining the accessible and non-accessible volumes is the use of very high pressure adsorption data as discussed by Malbrunot et al. (1997), who carried out experiments up to 500 MPa. These authors suggested that “*the ideal method according to the Gibbs surface definition would be to measure the adsorbent density for each gas with the gas itself, but this may not be practical*.” While they recognise that this approach is impractical (it would imply measuring isotherms up to 500 MPa or similar pressures), it should be clear that this method would lead to accessible volumes that are dependent on the guest molecule and therefore not valid for gas mixtures. Furthermore, even the accessible volume defined by this method will still yield a negative excess isotherm as shown by Eq. (14), since the negative excess at infinite pressure is obtained regardless of the porosity, ${{\varepsilon }_{m}}$, that one defines from the accessible volume. At best the excess amount at infinite pressure can only be exactly 0 if one defined the accessible volume, not as the true accessible volume but as the larger volume that will give the same close packing density at infinite pressure, ie $\frac{\eta _{CP}^{A}}{{{\eta }_{CP}}}=1$, but this would be a very arbitrary definition and it would again incur severe complications if mixtures of differently sized molecules are considered.

**Fig. 1:**
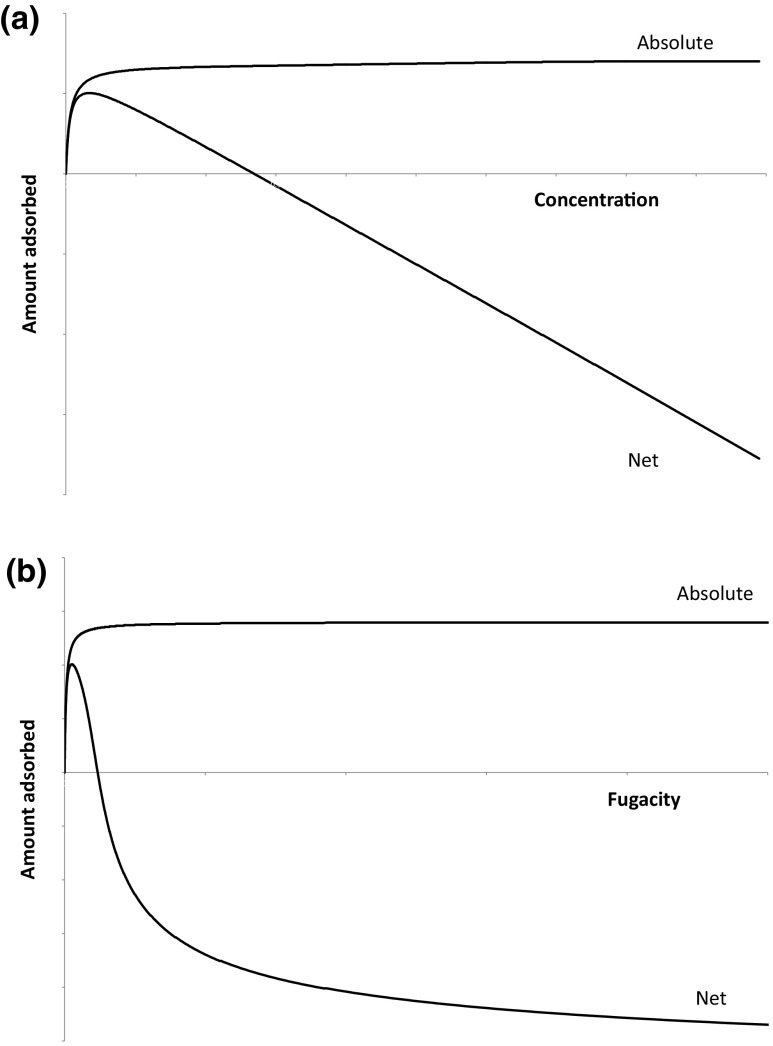
Comparison of absolute and net adsorption versus **a** concentration and **b** fugacity

**Fig. 2:**
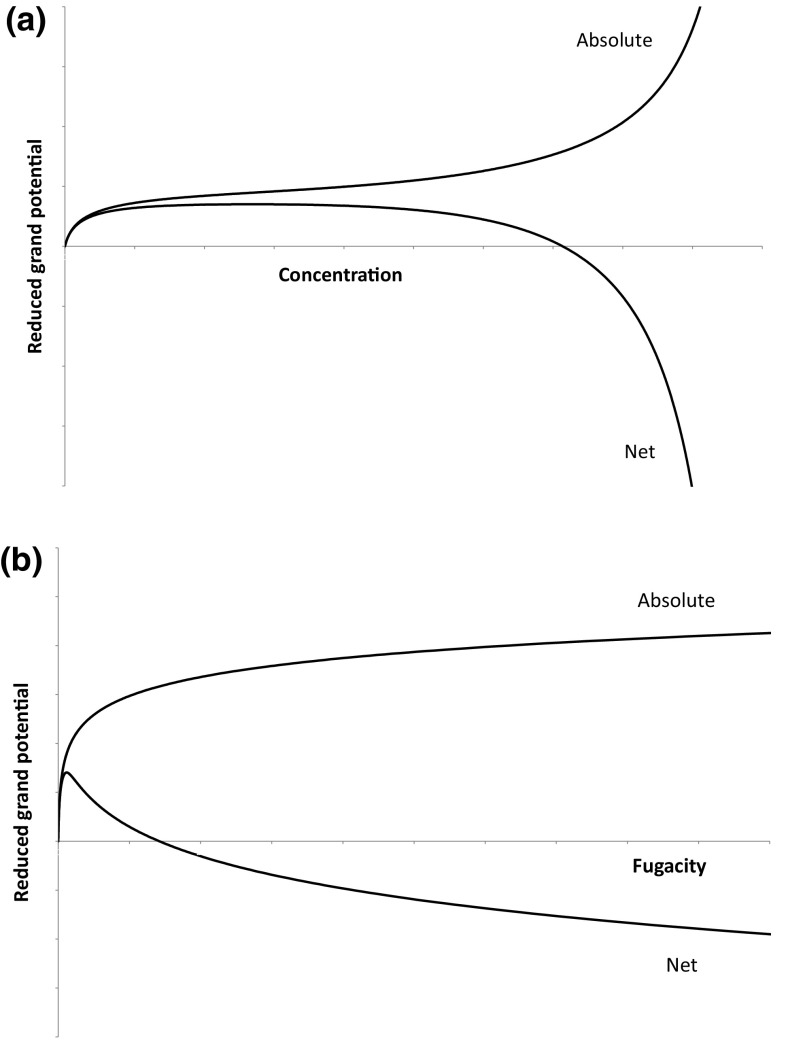
Comparison of reduced grand potentials defined in terms of absolute and net adsorption versus **a** concentration and **b** fugacity

**Fig. 3:**
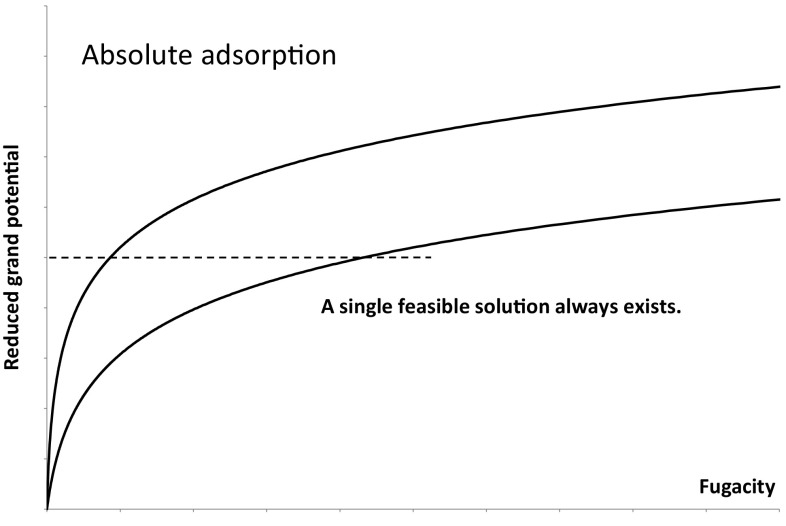
Absolute adsorption reduced grand potentials for two components with a selectivity of 5

**Fig. 4:**
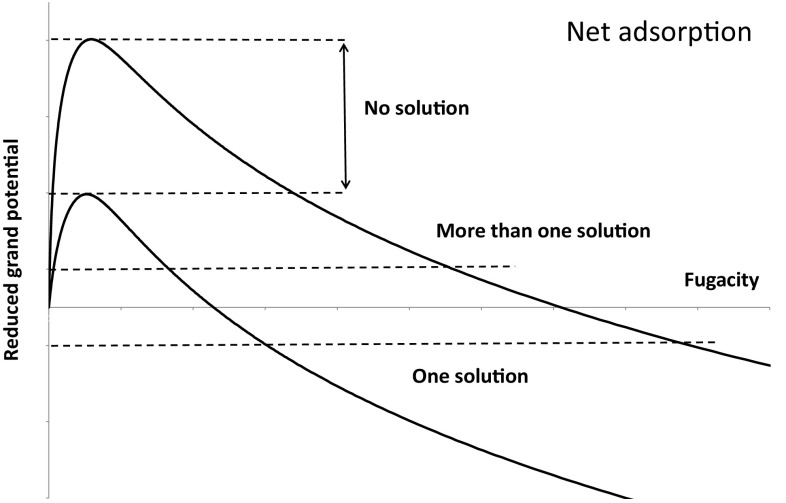
Net adsorption reduced grand potentials for two components with a selectivity of 5

This simple analysis shows that while there is always only one value of the absolute adsorbed amount corresponding to a pressure or fugacity, both net and excess adsorbed amounts may have two corresponding pressure or fugacity values. Figure 1 shows this qualitative behaviour, with excess adsorption omitted as it will be qualitatively similar to net adsorption, but will lie somewhere between the two curves shown. For completeness the parameters used to generate Figs. 1, 2, 3 and 4 are given in the Supplementary Information to this paper.

The fact that there is always one fugacity which corresponds to a given adsorbed amount already points to the fact that the natural thermodynamic variable to choose is the absolute adsorbed amount, also in view of the fact that numerical algorithms for the solution of multicomponent adsorption equilibrium are robust for the monotonically increasing absolute adsorption (Mangano et al. 2014).

## The ideal adsorbed solution theory (IAST) equations

The basic equations for the IAST were derived originally by Myers and Prausnitz (1965). The set of equations to be solved in terms of fugacities, which can be obtained from the expressions in Santori et al. (2015) assuming an ideal adsorbed solution, can be summarised as follows:

15 $$\phi_{i}{{y}_{i}}P=f_{i}^{0}\left( \text{ }\!\!\Psi\!\!\text{ } \right){{x}_{i}}~~~~~~~~~~~~~~~~~~~~~~~~~~~~~~~~~~~~~~~~~~~~~~~~~~~~~~~~~~~~~~~~i=1,~2\ldots Nc$$

These are the equilibrium relationships for each adsorbed component, where $f_{i}^{0}$ is the fugacity at which each pure component is at the same reduced grand potential, $\text{ }\!\!\Psi\!\!\text{ }$, and temperature of the mixture. The reduced grand potential is defined by the Gibbs adsorption isotherm

16 $${\Psi } = {\Psi }_{i} = \mathop \smallint \limits_{0}^{{f_{i} }} q_{i}^{0} \left( f \right)dlnf \qquad i = 1,~2 \ldots Nc$$

To close the problem, the total number of adsorbed moles can be found assuming zero mass or volume change upon adsorption

17 $$\frac{1}{{{q}_{t}}}=\underset{i=1}{\overset{Nc}{\mathop \sum }}\,\frac{{{x}_{i}}}{q_{i}^{0}\left( f_{i}^{0} \right)}~$$

Myers and Monson (2014) arrive at the commonly adopted formulation where $q_{i}^{0}=q_{i}^{abs}$, while Talu (2013) arrives at the same equations but $q_{i}^{0}=q_{i}^{net}$.

Recently Furmaniak et al. (2015) pointed out the fact that Talu’s approach leads to a reduced grand potential that has a maximum above a certain pressure. This can be understood considering the fact that once the net adsorbed amount becomes negative the integral equation, Eq. 16, will reach a maximum. The fact that there is not a one-to-one mapping of fugacity and reduced grand potential led them to abandon further testing of the IAST based on net adsorption (Talu 2013), since it would not be clear which root of Eq. 16  one should use. Figure 2 shows the qualitative shape of the reduced grand potential calculated using absolute and net adsorbed amounts. This shows that the limits at infinite pressure are opposite, the absolute adsorption value is +∞, while the net adsorption value is −∞. Again qualitatively a formulation based on the excess adsorbed amounts in Eqs. 15, 16, 17 would yield results similar to those of net adsorption.

The issues associated with the solution of Eq. 16 are made clearer if we consider Figs. 3 and 4 which show the reduced grand potentials calculated using a Langmuir adsorption isotherm for two components with a selectivity of five having the same saturation capacity for thermodynamic consistency (Ruthven 1984). In the absolute adsorption framework, Fig. 3, one can always find a solution, which is represented by a horizontal line parallel to the fugacity axis, i.e. the line for which the reduced grand potentials are equal, because for both components the reduced grand potentials will go to infinity at infinite pressure.

In the net adsorption framework, Fig. 4, there are regions where one can find a solution, which is not unique as pointed out by Furmaniak et al. (2015). The net adsorption framework will yield one feasible solution in a mixture for positive reduced grand potentials up to the maximum corresponding to the more weakly adsorbed component. Above this point, given that at a fixed fugacity the mixture grand potential will be an interpolation between the pure component grand potentials (Mangano et al. 2014), there is also a region between the two maxima where there is no solution. For negative values of the reduced grand potential there is only one solution, but this is physically impossible as the corresponding fugacity of the more weakly adsorbed component is smaller than that of the more strongly adsorbed species. Clearly also the solutions at a fugacity higher than the one corresponding to the maximum for the more strongly adsorbed component will be physically impossible since the solution will give a lower reference fugacity for the less strongly adsorbed components.

**Fig. 5:**
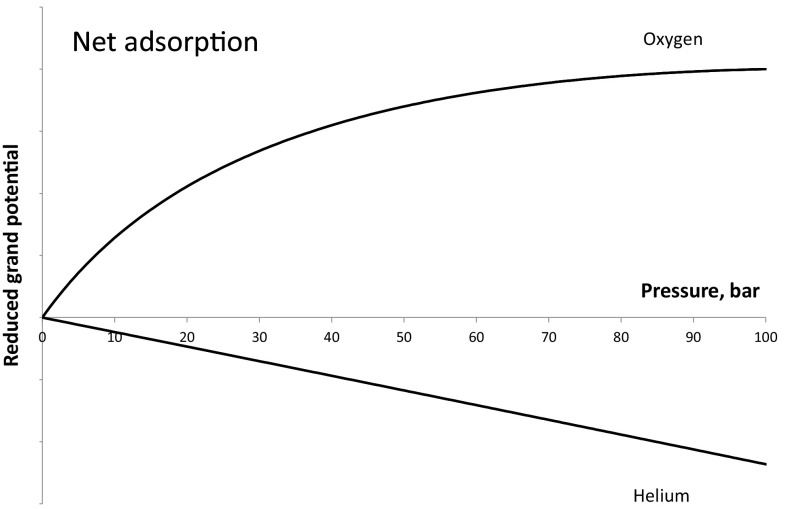
Net adsorption reduced grand potentials for oxygen and helium on 5A zeolite at 296 K. Parameters used to calculate the curves are given in Table 1

The region of no solution is not a purely hypothetical case. As a simple practical example one could consider the breakthrough curve for oxygen on 5A zeolite using helium as the carrier gas. While one can argue that in this case a good approximation is obtained assuming that helium is not adsorbed, as demonstrated by Brandani et al. (2016) the error of making this assumption is approximately 3% and not negligible because oxygen is not strongly adsorbed. In addition to this, a general and rigorous thermodynamic model should be applicable to any binary mixture. In this case at low pressures the net adsorption of oxygen is positive given that Ruthven and Xu (1993) report a dimensionless Henry law constant of 14.6 at 303 K, while helium adsorption is not zero, but the net adsorbed amount is negative given that at room temperature helium pycnometry is used to measure the skeletal density of microporous materials, i.e. the dimensionless Henry law constant is close to the porosity and clearly less than one. This is a system for which at low pressures one would expect the IAST to provide accurate predictions, but the formulation based on the net adsorption framework is not applicable because at all conditions the reduced grand potential of helium is negative and there are no solutions in the region 0–100 bar as can be seen in Fig. 5.

**Table 1:** Parameters used to calculate curves in Fig. 5

Parameter	${{q}_{i}}={{q}_{S}}\frac{{{b}_{i}}f}{1+{{b}_{i}}f}$	Source
*q*_*S*_	4.03 (mol kg^−1^)	Mathias et al. (1996). Assumed single site Langmuir as heat of adsorption of O_2_ is independent of loading
b_Oxy_	0.048 (bar^−1^)	Fit of data at 296 K from Talu et al. (1996)
b_He_	0.0013 (bar^−1^)	Assumed dimensionless Henry law constant of 0.42 (approximate porosity) and a value of 15 for O_2_ from Ruthven and Xu (1993). The ideal selectivity is 36:1 for O_2_/He. For helium the fugacity coefficient is always approximately 1, i.e.$f=P$
${{\rho }_{S}}$	1420 (kg m^−3^)	Estimated from crystal density as reported by First et al. (2011)

If one is still not convinced by these simple arguments, there is further clear evidence that the IAST formulated in the net adsorption framework is not correct. From a simple inspection of Eq. 17, one can see that the total amount adsorbed cannot be defined if the net amount adsorbed of one of the components in the mixture at the reference state is zero. The first occurrence would correspond to the maximum of the reduced grand potential for the weakest component in the mixture.

While these simple arguments are sufficient to understand that net adsorption cannot be used in a thermodynamic framework to arrive at an alternative formulation of the IAST, we still need to understand what caused the difference and demonstrate that even starting from the viewpoint of net adsorption one should arrive at the same equations as Myers and Monson (2014).

## Derivation of the Gibbs adsorption isotherm and the IAST

The key thermodynamic quantity in adsorption equilibria is the reduced grand potential and the corresponding Gibbs adsorption isotherm (Talu 2013; Myers and Monson 2014). Myers and Monson (2014) use the absolute adsorption and arrive at the classical result (see for example Ruthven 1984)

18 $$\text{ }\!\!\Psi\!\!\text{ }=-\frac{{{\mu }_{S}}-\mu _{S}^{0}}{RT}=\underset{0}{\overset{P}{\mathop \int }}\,\underset{i}{\mathop \sum }\,q_{i}^{A}dln{{f}_{i}}~~~~~~$$

where $\mu _{S}^{0}$ is the chemical potential of the solid on a volume basis in the absence of adsorbate.

Talu (2013) constructs a thermodynamic framework based on net adsorption and arrives at what is apparently a different definition

19 $${{\text{ }\!\!\Psi\!\!\text{ }}^{net}}=-\frac{{{\mu }_{S}}-{{\mu }_{S}}\left( P=0 \right)}{RT}=\underset{0}{\overset{P}{\mathop \int }}\,\underset{i}{\mathop \sum }\,q_{i}^{net}dln{{f}_{i}}~~~~~~$$

where ${{\mu }_{S}}\left( P=0 \right)$ is the chemical potential of the solid at zero pressure. Clearly

20 $${{\mu }_{S}}\left( P=0 \right)=\mu _{S}^{0}(P=0)$$

Both Talu (2013) and Myers and Monson (2014) start their respective definitions from the definition of the internal energy, but then Myers and Monson (2014) opt to use the Helmholtz energy as the potential in the solid phase and the Gibbs energy for the fluid phase, correctly indicating that what is being neglected is not important at low pressures. Given that the equilibrium between two phases is established when the chemical potential is the same for each component in both phases, it should be natural to formulate the equilibrium problem using the Gibbs energy for both phases. Talu (2013) invokes a series of thermodynamic relationships and derives the Gibbs–Duhem equation and from this the Gibbs adsorption isotherm.

An unambiguous derivation of the Gibbs adsorption isotherm for both net adsorption and absolute adsorption frameworks is needed in order to resolve this fundamental issue and understand the origin of the apparent discrepancy.

We start with the classical solution thermodynamics approach (Ruthven 1984) and simply write the total Gibbs energy of the system and use for the solid variables on a volume basis. The equivalent relationships on a mass basis are obtained using *M*_*S*_ when multiplying the chemical potential of the solid and result simply in a change in the units of the variables and are interchangeable if the solid density which includes the micropores, ${{\rho }_{S}}$, is known (Brandani et al. 2016).

21 $$G=\underset{i}{\mathop \sum }\,n_{i}^{A}\mu _{i}^{A}+{{V}_{S}}{{\mu }_{S}}$$

where $$n_{i}^{A}=q_{i}^{A}{{V}_{S}}$$.

If we are considering that the adsorbed phase is at equilibrium with a fluid phase we can also write

22 $$\mu _{i}^{A}={{\mu }_{i}}$$

The differential of the Gibbs energy is given by

23 $$dG=-SdT+VdP+\underset{i}{\mathop \sum }\,\mu _{i}^{A}dn_{i}^{A}+{{\mu }_{S}}d{{V}_{S}}$$

From the total differential of the Gibbs energy we obtain the Gibbs–Duhem equation

24 $$0=-SdT+VdP-\underset{i}{\mathop \sum }\,n_{i}^{A}d\mu _{i}^{A}-{{V}_{S}}d{{\mu }_{S}}$$

To define absolute adsorption we need to define the state of the solid without adsorbate

25 $$G^{0} = V_{S} \mu _{S}^{0}$$

and

26 $$d{{G}^{0}}=-{{S}^{0}}dT+{{V}^{0}}dP+\mu _{S}^{0}d{{V}_{S}}~~~~~~$$

and the corresponding Gibbs–Duhem equation

27 $$0 = - S^{0} dT + V^{0} dP - V_{S} d\mu _{S}^{0}$$

If we assume constant temperature and subtract the two Gibbs–Duhem relationships we obtain

28 $${{V}_{S}}d\left( {{\mu }_{S}}-\mu _{S}^{0} \right)=\left( V-{{V}^{0}} \right)dP-\underset{i}{\mathop \sum }\,n_{i}^{A}d\mu _{i}^{A}$$

We now introduce the additional assumption that in the system the quantity of solid does not change and hence we are considering a system of constant volume, ie V = V^0^ = V_S_.

If we now recall that for a fluid at constant temperature

29 $$d\mu _{i} = RTdlnf_{i}$$

we finally can arrive at

30 $$- \frac{{\mu _{S} - \mu _{S}^{0} }}{{RT}} = \mathop \int \limits_{0}^{P} \mathop \sum \limits_{i} \frac{{n_{i}^{A} }}{{V_{S} }}dlnf_{i}$$

which is the result obtained by Myers and Monson (2014) with a slightly different derivation.

We now proceed in the net adsorption framework. For this now we have to replace the solid with an identical volume filled with fluid which would be at equilibrium with the adsorbed phase. In this case we have

31 $$G^{{Fluid}} = \mathop \sum \limits_{i} n_{i}^{G} \mu _{i}$$

32 $$d{{G}^{Fluid}}=-{{S}^{Fluid}}dT+{{V}_{S}}dP+\underset{i}{\mathop \sum }\,{{\mu }_{i}}dn_{i}^{G}$$

and the corresponding Gibbs–Duhem equation

33 $$0=-{{S}^{Fluid}}dT+{{V}_{S}}dP-\underset{i}{\mathop \sum }\,n_{i}^{G}d{{\mu }_{i}}$$

At constant temperature, if we subtract the two Gibbs–Duhem relationships we now obtain

34 $${{V}_{S}}d{{\mu }_{S}}=\left( V-{{V}_{S}} \right)dP-\underset{i}{\mathop \sum }\,n_{i}^{A}d\mu _{i}^{A}+\underset{i}{\mathop \sum }\,n_{i}^{G}d{{\mu }_{i}}$$

and by integration

35 $$-{{V}_{S}}\frac{{{\mu }_{S}}-{{\mu }_{S}}\left( P=0 \right)}{RT}=\underset{0}{\overset{P}{\mathop \int }}\,\underset{i}{\mathop \sum }\,\left( n_{i}^{A}-n_{i}^{G} \right)dln{{f}_{i}}=\underset{0}{\overset{P}{\mathop \int }}\,\underset{i}{\mathop \sum }\,n_{i}^{net}dln{{f}_{i}}$$

This is the result obtained by Talu (2013), but it is not for the same thermodynamic function.

This issue is easily resolved if we define the reduced grand potential consistently as the difference between the chemical potential of the solid and the chemical potential of the solid without adsorbate, but at the same temperature and pressure of the system, since from Eq. 33 at constant temperature

36 $$\underset{i}{\mathop \sum }\,n_{i}^{G}d{{\mu }_{i}}={{V}_{S}}dP$$

then

37 $$- V_{S} \frac{{\mu _{S} - \mu _{S}^{0} }}{{RT}} = \mathop \int \limits_{0}^{P} \mathop \sum \limits_{i} \left( {n_{i}^{A} - n_{i}^{G} } \right)dlnf_{i} - \mathop \int \limits_{0}^{P} \frac{{V_{S} }}{{RT}}dP = \mathop \int \limits_{0}^{P} \mathop \sum \limits_{i} n_{i}^{A} dlnf_{i}$$

which is the classical result and shows that absolute adsorption is the fundamental thermodynamic variable in adsorption thermodynamics. Since equilibrium requires that the two phases should be at the same pressure if surface effects are neglected (Prausnitz et al. 1999), ie the classical solution thermodynamics approach which is at the basis of the IAST, the grand potential should not be defined relative to zero pressure.

A rigorous definition of the reference state would appear to require the use of a Poynting correction (Prausnitz et al. 1999) in Eq. 15 in order to take into account the difference between the pressure of the reference state for the pure adsorbate and the pressure of the mixture. This is in fact negligible because for a single adsorbate the change in chemical potential due to a change in pressure at constant temperature and constant amount adsorbed combining Eqs. 24 and 27 is

38 $${{\Delta }}\mu _{{}}^{A} = \frac{{V_{S} }}{{n_{{}}^{A} }}\left( {{{\Delta }}P - {{\Delta }}\mu _{S} } \right) = - \frac{{V_{S} }}{{n_{{}}^{A} }}{{\Delta }}\left( {\mu _{S} - \mu _{S}^{0} } \right) \approx 0$$

If one assumes that the change in chemical potential of the solid due to a change in the external pressure is not affected by the presence of the adsorbate, which is reasonable for a rigid crystal, then the RHS of Eq. 38 is zero. Even if this is not exactly true, one can see that the resulting Poynting correction factor would be very small given that it is calculated from the difference of $\text{ }\!\!\Delta\!\!\text{ }P$ and $\text{ }\!\!\Delta\!\!\text{ }{{\mu }_{S}}$ and not $\text{ }\!\!\Delta\!\!\text{ }P$ alone as for a normal liquid phase (Prausnitz et al. 1999).

This is the reason why one can use the Helmholtz energy for the adsorbed phase in the derivation of the IAST (Myers and Monson 2014) and the resulting formulation, Eqs. 14 and 15, is not limited to low pressures if fugacities are used to correct for the gas phase non-ideality.

The final step for the closure of the equations of the IAST is the derivation of the total amount adsorbed in an ideal adsorbed mixture. The differential of the grand potential at constant temperature for the mixture is given by

39 $$- V_{S} d\left( {\mu _{S} - \mu _{S}^{0} } \right) = \mathop \sum \limits_{i} n_{i}^{A} d\mu _{i}^{A}$$

which can also be written for the pure component

40 $$- V_{S} d\left( {\mu _{S} - \mu _{S}^{0} } \right) = n_{i}^{{A0}} d\mu _{i}^{{A0}}$$

41 $$\frac{{ - V_{S} }}{{n_{i}^{{A0}} }} = \frac{{d\mu _{i}^{{A0}} }}{{d\left( {\mu _{S} - \mu _{S}^{0} } \right)}}$$

Since the reference state is chosen as that of the pure component at the same temperature and grand potential of the mixture and given that the adsorbed phase is ideal

42 $$\frac{{d\upmu _{i}^{{A0}} }}{{d\left( {\upmu _{S} - \upmu _{S}^{0} } \right)}} = \frac{{d\upmu _{i}^{A} }}{{d\left( {\upmu _{S} - \upmu _{S}^{0} } \right)}}$$

and substituting into Eq. 39 we have

43 $$1=\underset{i}{\mathop \sum }\,\frac{n_{i}^{A}}{n_{i}^{A0}}~~~~~$$

or (Myers and Monson 2014)

44 $$\frac{1}{n_{Tot}^{A}}=\underset{i}{\mathop \sum }\,\frac{x_{i}^{{}}}{n_{i}^{A0}}~~~~~$$

Talu (2013) using the incorrect reduced grand potential arrives at a similar expression in terms of net adsorption

45 $$\frac{1}{n_{Tot}^{net}}=\underset{i}{\mathop \sum }\,\frac{x_{i}^{net}}{n_{i}^{net0}}~~~~~$$

but this final expression is clearly incorrect since it is undefined at higher pressures which correspond to the net adsorption of the pure components being zero.

## Conclusions

In the formulation of a thermodynamic framework for mixed gas adsorption we have shown that only the absolute adsorbed amount has a one to one correspondence between fugacity and adsorbed amounts. Both net and excess adsorption will initially increase linearly and then go through a maximum and finally become negative if pressure (or fugacity) is sufficiently high. This leads to the important conclusion that it is not possible to develop a rigorous version of the IAST based on either the net or the excess adsorbed amounts.

Clearly if net and excess adsorbed amounts are used to approximate absolute adsorption, then values obtained using excess adsorption will be closer to the true solution, but the correct approach to follow is to define the solid volumes as discussed in Brandani et al. (2016) and carry out the predictions directly using absolute adsorbed amounts.

Having established on qualitatitive grounds that the definition of the IAST equations obtained by Talu (2013) are inconsistent, we have proceeded to prove that the key issue in Talu’s derivation is the fact that the equilibrium between the phases is not defined at the same pressure, which is the starting point of classical fluid phase equilibria formulations. This small inconsistency leads to a set of equations which is incorrect, does not have unique solutions and is undefined when the lighter components reach the conditions where net adsorption is zero. When the correct reference state is used, the original IAST formulation is recovered whether one starts from absolute or net adsorbed amounts, arriving at the conclusion that there is only one definition of ideal adsorbed solution.

The analysis presented is a further indication that absolute adsorption is the thermodynamic variable to use in describing adsorption. While net and excess adsorption can be used to report experimental results, it is still necessary to determine the density of the microporous solid, which includes the micropores (Brandani et al. 2016), in order to be able to use the data in adsorption process simulations and consistent thermodynamic frameworks for mixed gas adsorption.

## Electronic supplementary material

Below is the link to the electronic supplementary material.

Supplementary material 1 (DOCX 27 KB)

## List of symbols

$c_{i}$: Gas phase concentration of component i (mol m^–3^)
$c_{i}^{\infty }$: Concentration of component i at infinite pressure (mol m^–3^)
$f_{i}$: Fugacity of component i in gas mixture (kPa)
$f_{i}^{0}$: Fugacity of pure component i at the reference state (kPa)
$$G$$: Gibbs energy (J)
${\text{G}}^{0}$: Gibbs energy of the solid without the adsorbate (J)
$K$: Dimensionless Henry law constant (–)
*M*_*S*_: Mass of solid (kg)
$n_{i}^{A}$: Moles adsorbed of component i (mol)
$n_{i}^{abs}$: Absolute adsorbed amount of component i (mol)
$n_{i}^{ex}$: Excess adsorbed amount of component i (mol)
$n_{i}^{net}$: Net adsorbed amount (mol)
$n_{S}^{{}}$: Moles of solid (mol)
$n_{Tot}$: Total moles in the system (mol)
$$P$$: Pressure (kPa)
$q_{i}^{A}$: Adsorbed phase concentration of component i (mol m^–3^)
$q_{i}^{abs}$: Absolute adsorbed concentration of component i (mol m^–3^)
$q_{i}^{net}$: Net adsorbed concentration of component i (mol m^–3^)
$q_{t}$: Total adsorbed phase concentration (mol m^–3^)
$R$: Ideal gas constant (J mol^–1^ K^–1^)
$$S$$: Entropy (J K^–1^)
$$T$$: Temperature (K)
$V_{NA}$: Volume not accessible (m^3^)
$V_{S}$: Volume of solid, including micropores (m^3^)
$x_{i}^{{}}$: Mole fraction in adsorbed phase (–)
$y_{i}^{{}}$: Mole fraction in gas phase (–)
$$z$$: Compressibility factor (–)

## Greek letters

$\phi_{i}$: Fugacity coefficient of component i in gas mixture (–)
$\mu_{i}^{{}}$: Gas phase chemical potential of component i (J mol^–1^)
$\mu_{i}^{A}$: Chemical potential of adsorbate i (J mol^–1^)
$\mu_{S}$: Chemical potential of solid (volume basis) (J m^–3^)
$\mu_{S}^{0}$: Chemical potential of solid without adsorbate (J m^–3^)
$\eta_{CP}$: Reduced density at close packing (–)
$\eta_{CP}^{A}$: Reduced density of adsorbed phase at close packing (–)
$\rho_{S}$: Solid density including micropores (kg m^–3^)
${\Psi}$: Reduced grand potential (mol m^–3^)
