# Supplementary material for: Net, excess and absolute adsorption in mixed gas adsorption
Source: Adsorption (Boston). 2017 Feb 24;23(4):569–76. doi: 10.1007/s10450-017-9875-4 (PMC7010369; doi:10.1007/s10450-017-9875-4)
Supplement: Supplementary file 1 — Supplementary material 1 (DOCX 27 KB) [file 10450_2017_9875_MOESM1_ESM.docx]

**Net, Excess and Absolute Adsorption in Mixed Gas Adsorption**

**Supplementary Information**

Stefano Brandani*, Enzo Mangano and Mauro Luberti

Scottish Carbon Capture and Storage

School of Engineering, The University Edinburgh,

The King’s Buildings, Mayfield Road, Edinburgh EH9 3FB

United Kingdom

**Details of calculations in Figures 1-4**

These figures are presented to give the qualitative behaviour and observe relevant trends, therefore the parameters used do not represent a specific system.

The gas phase is described using the Redlich-Kwong equation of state (EOS) with the compressibility factor given by

$z=\frac{P}{cRT}=\frac{1}{1-\eta}-\frac{\epsilon\sqrt{\frac{T_{C}}{T}}}{RT}\frac{\eta}{1+\eta}$ (SI-1)

where $\eta=bc$ and the EOS parameters are related to the critical point by:

$b=0.08664\frac{RT_{C}}{P_{C}}$ and $\epsilon=\frac{0.4278}{0.08664}RT_{C}$ (SI-2)

At infinite pressure $\eta=1$.

The fugacity coefficient is given by

$ln\phi=ln\left( \frac{1}{1-\eta} \right)+ \frac{\epsilon\sqrt{\frac{T_{C}}{T}}}{RT} ln\left( \frac{1}{1+\eta} \right)+z-1-lnz$ (SI-3)

The Langmuir adsorption isotherm expressed in terms of fugacity is

$q^{A}=q_{S}\frac{b_{L}f}{1+b_{L}f}$ (SI-4)

where the fugacity is given by $f=\phi P$.

In the calculations for Figures 1-4 $q_{S}=\frac{0.28}{b}$, where $\frac{\eta_{CP}^{A}}{\eta_{CP}}\varepsilon_{m}=0.28$. For the fluid $T_{C}=200$K and $P_{C}=40$ bar. Finally $b_{L}=0.1$ bar^–1^.

For Figs 3-4 the second component is calculated using $b_{L}=0.5$ bar^–1^, i.e. an ideal selectivity of 5.
